# The Mediating Effects of Social Networks and Wisdom on the Relationship between Lifestyle Habits and Healthy Aging in Older Adults with Chronic Diseases

**DOI:** 10.3390/bs13080688

**Published:** 2023-08-18

**Authors:** Hee-Kyung Kim, Hye-Suk Oh, Cheol-Hee Park

**Affiliations:** 1 Department of Nursing, Kongju National University, Gongju 32588, Republic of Korea; 2 Department of Nursing, Graduate School, Kongju National University, Gongju 32588, Republic of Korea; d20230184@smail.kongju.ac.kr (H.-S.O.); cheolhee33@smail.kongju.ac.kr (C.-H.P.)

**Keywords:** elderly, healthy aging, lifestyle habits, social networks, wisdom, chronic disease

## Abstract

Background: The purpose of this study was to analyze the mediating effects of social networks and wisdom on the relationship between lifestyle habits and healthy aging in older adults with chronic diseases. Methods: Participants were 120 community-dwelling older adults aged 65 and older with at least one chronic disease. Data were collected from elderly people by visiting nursing care worker training centers, senior centers, social gatherings, and home welfare centers in D, G, and S cities, with a questionnaire of lifestyle habits, social networks, wisdom and healthy aging, and general characteristics. Results: There were differences in the degree of healthy aging according to age (F = 3.76, *p =* 0.026), spousal relationship (t = 3.11, *p* = 0.002), education (F = 9.08, *p* < 0.001), number of diseases (F = 8.65, *p* < 0.001), and economic level (t = −2.45, *p* = 0.016). The most common diseases among the subjects were hypertension, hyperlipidemia, joint diseases and diabetes mellitus. Social networks (β = 0.46, *p <* 0.001) and wisdom (β = 0.55, *p <* 0.001) had partial mediating effects (z = 4.15, *p <* 0.001; z = 5.11, *p <* 0.001) on the relationship between subjects’ lifestyle habits and healthy aging. Conclusions: To increase the degree of healthy aging of subjects, it is necessary to establish a mediating intervention program that manages to have good lifestyle habits in daily life, increase social networks, and become wise.

## 1. Introduction

The World Health Organization (WHO) takes a multidimensional approach to human aging from the perspective of healthy aging [1]. Healthy aging refers to a low level of psychological, physical, and social aging, and it is the total interaction of psychological, physical, and social factors that results in attitudes toward aging, which is a major factor in determining the overall quality of life of older adults [2]. Therefore, since healthy aging is a set of processes and practical concepts for the elderly to achieve successful aging as they go through life [3], the elderly should prepare for a healthy old age by engaging in healthy and desirable daily activities to reduce psychological, physical, and social aging. In the past, various studies on the health of the elderly have generally been conducted with terms such as successful aging, quality of life, health-related quality of life, and health preservation, but it is more appropriate to study healthy aging by viewing the life of the elderly as a process [4].

In 2022, the elderly population in South Korea will account for 17.5% of the total population, and by 2025, it will account for 20.6%, entering the super-elderly society. As Korea is moving from an aging society to an ultra-elderly society faster than other Organization for Economic Co-operation and Development (OECD) countries [5], the health and well-being of the rapidly growing elderly population require much attention and support from professionals. Healthy aging is the maintenance of high levels of well-being in the domains of objective and subjective physical and cognitive health, active living, life satisfaction, and social support [6] and optimal well-being overall [7]. Maintaining these conditions can lead to a comfortable life and successful aging in place. However, according to the National Survey of the Elderly, more than 75% of the elderly have two or more chronic diseases, and these conditions require ongoing treatment and management [8]. As people age, they experience health problems, such as pain from chronic diseases, limited activities of daily living, social difficulties, and loneliness, so they often rate their own health as fair or poor. Moreover, the older people are, the more likely they are to perceive themselves as unhealthy [9], so interventions to help older people manage their health are urgently needed. 

First of all, when it comes to healthy aging in the elderly, daily lifestyle habits are very important. Lifestyles are decisions made by individuals that affect health and refer to things that can be controlled to some extent [10]. Healthy lifestyles among the elderly reduce the occurrence of chronic diseases and depression, as well as extend life expectancy [11]. Your lifestyle habits have a huge impact on your health. In particular, various daily habits are continuous and repeated behaviors, so they refer to all health-related behaviors that people perform in their daily lives, whether consciously or unconsciously, such as exercise and activity, diet, alcohol consumption, stress management, rest, and sleep [10,12]. According to the 2022 Senior Citizen Statistics, walking, aerobic physical activity, and strength training are the most common types of exercise among the elderly, but the rate of habitual exercise is very low [5], and there is a lack of specific reports on other types of lifestyle habits, so it is necessary to investigate them. In particular, poor lifestyle habits have a negative impact on health and life [12], so it is necessary to manage to maintain good lifestyle habits for healthy aging.

In a study by Lee and Kim [13], the more appropriate the lifestyle habits and the larger the social networks of the elderly, the better their health was preserved, and lifestyle habits and social networks were found to be the main influencing factors for health preservation, so it is desirable to consider the effect of social networks when investigating lifestyle habits and healthy aging. A study of the social networks of the elderly found that more than half of the elderly reported that they had 1–2 people to call when they were sick or needed help around the house and that they were moderately satisfied with their family relationships but that they had fewer relationships with family members and acquaintances during the COVID-19 pandemic than before. In addition, compared to other age groups, the elderly have lower rates of participation in continuing education, social service, and social organizations, and they have fewer social gatherings due to COVID-19 [5]. Originally, social networks represented social contacts and were synonymous with social support. In particular, humans are social animals and live their lives through social relationships. Therefore, awareness of social networks is highly correlated with healthy lifestyle habits, leading to increased subjective well-being and better health preservation [13]. It also fulfills self-esteem and increases perceptions of safety [14] and can play a mediating role in the relationship between daily life and healthy aging in older adults [13].

And for the healthy aging of the elderly, it is necessary to live a wise life in daily life. Wisdom is an attitude of pursuing truth throughout life, cherishing artistic endeavors, and embodying morality and humanity. It emphasizes the value of the present moment [15]. This disposition contributes to resolving problems, conflicts, crises, and challenges faced in old age, subsequently influencing life satisfaction [16]. In a study involving middle-aged men, a positive correlation was found between lifestyle and wisdom. Wisdom also exhibited a complete mediating effect in the relationship between the health habits and health preservation of middle-aged men. This suggests the need for strategies to enhance wisdom [17]. Therefore, it becomes imperative to cultivate healthy lifestyles among the elderly, which in turn fosters wisdom and ultimately facilitates healthy aging. 

The wiser the elderly, the better they preserved their health [18]. Wisdom helps to overcome life’s difficulties by accepting both the positive and negative aspects of reality [19], which can lead to healthy aging. The wisdom of the elderly is gained from their numerous life experiences and makes them more patient, tolerant, and receptive to the emotions of others, leading to healthy and successful aging by integrating the dual meanings of aging, i.e., the negative and positive aspects [20,21,22,23]. Wisdom tends to increase in old age more than in other age groups because older adults have more time to transcend through self-reflection, self-examination, and self-awareness, and their life experiences increase their wisdom [21,24]. In addition, wisdom was a major influence on successful aging in older adults, and the subdomains of wisdom, empathic emotion, self-reflection, and life-completion experiences, led to successful aging [22,25]. Therefore, adding wisdom variables in the process of forming daily lifestyle habits can help the elderly achieve healthy old age, and wisdom may play a mediating role in the relationship between lifestyle habits and healthy aging. 

Therefore, this study aims to identify the relationship between lifestyle habits, social networks, and wisdom and healthy aging among community-dwelling elderly people with chronic diseases and to analyze the mediating effects of social networks and wisdom on the relationship between lifestyle habits and healthy aging to provide the basis for developing healthy aging intervention programs to help elderly people achieve healthy aging while maintaining good lifestyle habits. 

## 2. Materials and Methods

### 2.1. Research Design

**Figure 1:**
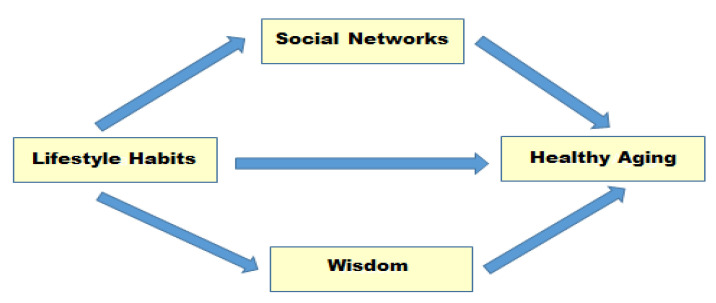
Research design.

Mediational effects are effects mediated by a third variable (M) on the effect of a predictor (X) on an outcome (Y). The existence of a mediator implies a causal pathway in which the predictor increases the mediator, and the increased mediator affects the dependent variable [26]. In other words, the mediating effect of social networks and wisdom in the elderly is that the better the lifestyle habits, the higher the degree of social networks and wisdom, and the increased social networks and wisdom increase healthy aging. Therefore, this study is a descriptive correlational study to analyze the mediating effect of social networks and wisdom in the relationship between lifestyle habits and healthy aging among community-dwelling elderly people with chronic diseases. The schematic of the study is shown in Figure 1.

### 2.2. Participants

Community-dwelling male or female older adults, 65 years of age or older, with at least one chronic medical condition, who have not been diagnosed with dementia by a physician, have a mild cognitive impairment, including memory, and are able to respond to the questionnaire and communicate. Exclusion criteria include elderly people diagnosed with moderate or severe dementia by a doctor, unable to communicate, unable to understand the contents of the questionnaire, or hospitalized in a hospital. The number of participants in the study was based on the study of Kim et al. [27], which used statistical analysis of stepwise multiple regression to study the mediating effect and the statistical program G-power3.1.9.7. is used to calculate the number of participants. As a result of the analysis, the sample size required to maintain three predictor variables, an effect size of 0.15, a significance level of 0.05, and a power of 0.95 was 119, and 132 questionnaires were prepared in consideration of a 10% dropout rate, but data were collected from the final 120 participants, and all 120 were used for the study. 

### 2.3. Procedures

After receiving approval from the university’s Institutional Review Board (IRB), researchers visited one nursing home caregiver training center in Sejong, one senior center in Gongju, one senior citizens’ fellowship group, one nursing home caregiver training center in Daejeon, and one home welfare center for 30 days from 24 May to 23 June 2023, and met with the directors, representatives, and meeting chairpersons to explain the purpose of the study and the questionnaire survey method, and distributed questionnaires to subjects who met the selection criteria after obtaining permission. If the subjects were willing to participate in the study and voluntarily agreed, they signed a written consent form and filled out the questionnaire. A total of 120 subjects completed the questionnaires, with the researchers reading the contents to them if necessary or answering questions. The results will be fed back to the participants afterward to increase their willingness to participate in the study. The questionnaire took about 15 min to complete. 

### 2.4. Measures

The instrument is a structured, self-completion questionnaire consisting of 10 general characteristics, 39 lifestyles, 15 social networks, 43 wisdom, and 20 healthy aging questions.

#### 2.4.1. Lifestyle Habits

We used the health promotion style measurement tool developed by Walker et al. [28], modified for Korean culture by Hur [29] and Cho [30], and the lifestyle measurement tool used by Kwon [10]. The instrument consists of 13 questions on eating habits, 3 questions on sleep and rest habits, 10 questions on stress management habits, 5 questions on drinking habits, and 8 questions on exercise habits. The total mean score ranges from 1 to 4 on a 4-point Likert scale from 1 for “never” to 4 for “always”, with higher scores indicating a healthier lifestyle. At the time of development, the reliability of dietary habits in Kwon’s [10] study was Cronbach’s α = 0.62, sleep and rest habits 0.74, stress management habits 0.74, drinking habits 0.67, and exercise habits 0.89, and the reliability of dietary habits in this study was Cronbach’s α = 0.70, sleep and rest habits 0.74, stress management habits 0.78, drinking habits 0.60, and exercise habits 0.87, and the overall reliability was 0.85. 

#### 2.4.2. Social Networks

The social network instrument for the elderly was the social network instrument developed by Lee [31]. It consists of 15 questions, including 5 questions on social participation activities, 5 questions on self-development activities, and 5 questions on activities at home. Each question is answered on a 5-point Likert scale, ranging from 1 for social participation activities to 5 for self-development activities and 5 for activities at home, from 1 for “not at all” to 5 for “very often”. Higher scores indicate higher levels of social connectedness. At the time of development, the reliability coefficient Cronbach’s α = 0.88 was 0.63 for social participation activities, 0.90 for self-development activities, and 0.72 for activities at home. In this study, the overall reliability was 0.86, with 0.64 for social participation activities, 0.93 for self-development activities, and 0.70 for home activities. 

#### 2.4.3. Wisdom

The Korean Wisdom Scale (KMWS) developed by Kim [32] was used (Appendix A, Table A1). It consists of 43 items, including 16 items on cognitive competence, 11 items on integrity and balance, 10 items on positive life attitude, and 6 items on empathetic interpersonal relationships. Each item is scored on a 5-point Likert scale ranging from 1, “not true at all”, to 5 “absolutely true”, with higher scores indicating higher levels of wisdom. At the time of development, the reliability of Cronbach’s α was 0.90 for cognitive competence, 0.83 for refinement and balance, 0.83 for positive life attitude, and 0.76 for empathic interpersonal relationships, with an overall reliability of 0.93. In this study, Cronbach’s α was 0.92 for cognitive competence, 0.91 for integrity and balance, 0.90 for positive life attitude, and 0.92 for empathic interpersonal relationships, for an overall reliability of 0.96. 

#### 2.4.4. Healthy Aging

The Healthy Aging Tool developed by Ko [33] was used. It consists of 20 questions: 6 questions on physical health, 6 questions on cognitive and mental health, and 8 questions on social support health. Each question is on a 5-point Likert scale ranging from 1, “not at all”, to 5 ”very much”. Higher scores indicate higher levels of healthy aging. At the time of tool development, the reliability coefficient Cronbach’s α = 0.89 was 0.61 for physical health, 0.86 for cognitive and mental health, and 0.88 for social support health. In this study, the overall reliability was 0.91, with 0.71 for physical health, 0.88 for cognitive and mental health, and 0.91 for social support health. 

### 2.5. Data Analysis

The SPSS^®^ Statistics 27.0 software made by IBM Corporation, Armonk, NY, USA, was used to analyze the general characteristics of the subjects and the degree of variables using descriptive statistics, such as real numbers, percentages, means, and standard deviations, and the differences in the degree of healthy aging according to the general characteristics of the elderly were analyzed by *t*-test, ANOVA, and post hoc analysis by Scheffe’s test. The correlations between lifestyle habits, social networks, wisdom, and healthy aging of the elderly were analyzed by Pearson’s correlation coefficients. The mediating effect of social network and wisdom in the relationship between lifestyle habits and healthy aging of the elderly was analyzed using the method of Baron, Kenny [26] through multiple regression analysis, and the adequacy of the mediating effect was confirmed by the Sobel test [34]. 

### 2.6. Ethical Consideration

The study was approved by the Institutional Review Board of K University (KNU_IRB_2023-48) and ensured compliance with ethical guidelines regarding the purpose, methods, and protection of participants’ rights. The consent form provided participants with information about anonymity and confidentiality. Subjects were informed that they could withdraw from the study at any time and would not be penalized. Personal information collected was managed in accordance with privacy laws, and the researchers endeavored to maintain the confidentiality of all information obtained. Informed consent was obtained from all participants, and the data collected were stored securely in a locked bookcase accessible only to the researchers for three years. Computerized data were anonymized for statistical analysis. After the study was completed, participants were informed that research-related materials would be kept for three years and then destroyed using a shredder.

## 3. Results

### 3.1. General Characteristics of Participants

The general characteristics of the study population were as follows (Table 1). Subjects were aged 65–88 years, with a mean age of 71.48 ± 6.77 years, with 55.0% (66 persons) aged 65–69 years. In terms of gender, 68.3% (82 persons) were female. Regarding the relationship with their spouse, 80.0% (96 persons) were living together, 51.7% (62 people) had graduated from middle school or high school, 50.0% (60 persons) were religious, 57.5% (69 persons) had no job, and 34.0% (20 persons) had other occupations. The average number of diseases per participant was 2.13, with 50.0% (60 persons) reporting one disease, and those with multiple responses were 61 persons for hypertension, 55 persons for hyperlipidemia, 40 persons for joint disease, and 24 persons for diabetes mellitus. The majority of respondents (80.0%, 96 persons) reported that their economic level was difficult or moderate. 

### 3.2. Differences in Healthy Aging According to General Characteristics of Participants

The differences in healthy aging according to the general characteristics of the subjects were as follows (Table 1). Age (F = 3.76, *p* = 0.026), spousal relationship (t = 3.11, *p* = 0.002), education (F = 9.08, *p* < 0.001), number of diseases (F = 8.65, *p* < 0.001), and economic level (t = −2.45, *p* = 0.016) showed differences in the degree of healthy aging at a statistically significant level. In other words, the elderly aged 65–69 years had a higher level of healthy aging than those aged 80 years or older; those living with a spouse had a higher level of healthy aging than those who were widowed, divorced, or separated; and those who graduated from college or higher had a higher level of healthy aging than those who graduated from elementary school or lower, middle school, or high school. The elderly with one disease had a higher degree of health aging than the elderly with four or more diseases. The elderly who perceived that the economic level was high had a higher degree of health aging than the elderly who perceived that the economic level was low or moderate. Other gender and religion. The differences in healthy aging by occupation were not statistically significant. 

### 3.3. Degree of Lifestyle Habits, Social Networks, Wisdom and Healthy Aging of Participants

The subjects’ lifestyle, social network, wisdom and health aging, and the degree of each subdomain were as follows (Table 2). Subjects’ lifestyle habits were 2.84 ± 0.32 out of 4. Among the subdomains, drinking habits scored 3.29 ± 0.45, eating habits scored 2.92 ± 0.40, and exercise habits scored the lowest at 2.60 ± 0.67. Social networking was 2.95 ± 0.69 out of 5, with the highest score of 3.09 ± 0.71 for household activities and the lowest score of 2.75 ± 0.85 for social participation activities. Wisdom was 3.45 ± 0.60 out of 5, with empathetic interpersonal relationships being the highest at 3.74 ± 0.72, refining and balancing being 3.52 ± 0.63, a positive attitude toward life being 3.50 ± 0.66 and cognitive competence being the lowest at 3.27 ± 0.71. Healthy aging was 3.56 ± 0.58 out of 5, with social support health being the highest at 3.62 ± 0.66 and cognitive-emotional and spiritual health being the lowest at 3.49 ± 0.77.

### 3.4. Correlations between Lifestyle Habits, Social Networks, Wisdom and Healthy Aging of Participants

The correlations between subjects’ lifestyle habits, social networks, wisdom and healthy aging were as follows (Table 3). Healthy aging was highly positively correlated with lifestyle habits (r = 0.63, *p <* 0.001), social networks (r = 0.65, *p <* 0.001), and wisdom (r = 0.73, *p <* 0.001). In other words, the better the lifestyle habits, the wider the social networks, and the higher the wisdom, the higher the healthy aging theft. 

### 3.5. Mediating Effect of Social Networks and Wisdom in the Relation between Lifestyle Habits and Healthy Aging of Participants

Before testing the mediating effect of social networks and wisdom on the relationship between subjects’ lifestyles and health aging, the assumptions of the regression analysis were tested. The residual plots were checked for equality of variance, and the Durbin–Watson value for independence of the residuals was 1.635, which is close to 2, satisfying the assumption of independence. To verify the normality of the error term, we checked the P-P plot for independence and found that it was normally distributed. In addition, the tolerance for multicollinearity among the independent variables was 0.50~0.83, and the Variance Inflation Factor (VIF) of the variables was 1.21–2.02, which is less than 10, so the basic assumptions of equality of variance and normal distribution of the residuals were satisfied. 

#### 3.5.1. Mediating Effect of Social Networks in the Relation between Lifestyle Habits and Healthy Aging of Participants

**Figure 2:**
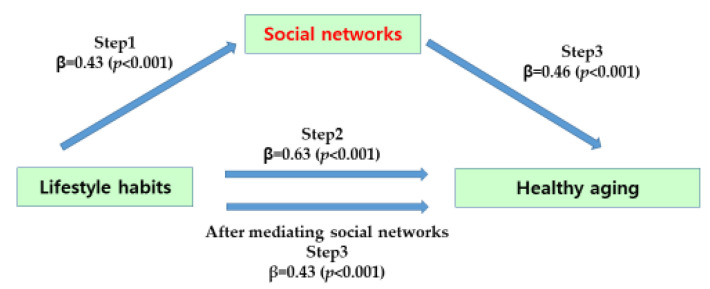
The mediating effect of social networks of participants.

First, we tested the mediating effect of social networks on the relationship between subjects’ lifestyles and healthy aging (Table 4). In step 1, the independent variable, lifestyle, had a statistically significant effect on the mediating social network (β = 0.43, *p <* 0.001), and in step 2, the independent variable, lifestyle, had a statistically significant effect on the dependent variable, health aging (β = 0.63, *p <* 0.001). In step 3, the independent variable, lifestyle, and the mediator, social network, were simultaneously entered into the regression model to predict healthy aging and social network had a significant effect on healthy aging (β = 0.46, *p <* 0.001). The regression coefficient of lifestyle on healthy aging decreased from 0.63 to 0.43 and was statistically significant (β = 0.43, *p <* 0.001). Therefore, it was found that social relationship networks had a partial mediating effect on the relationship between lifestyle and healthy aging, and the significance of the mediating effect was statistically significant using the Sobel test (Z = 4.15, *p <* 0.001) (Figure 2). 

#### 3.5.2. Mediating Effect of Wisdom in the Relation between Lifestyle Habits and Healthy Aging of Participants

**Figure 3:**
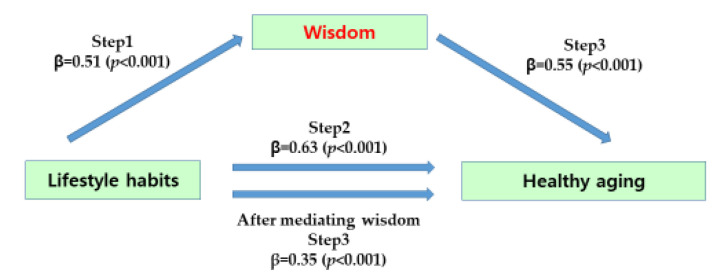
The mediating effect of the wisdom of participants.

First, we tested the mediating effect of wisdom on the relationship between subjects’ lifestyles and healthy aging, and the results are as follows (Table 5). In step 1, the independent variable, lifestyle, had a statistically significant effect on the mediating variable, wisdom (β = 0.51, *p <* 0.001), and in step 2, the independent variable, lifestyle, had a statistically significant effect on the dependent variable, healthy aging (β = 0.63, *p <* 0.001). In step 3, the independent variable, lifestyle, and the mediator, wisdom, were simultaneously entered into the regression model to predict healthy aging, and wisdom had a significant effect on healthy aging (β = 0.55, *p <* 0.001). The regression coefficient of lifestyle on healthy aging decreased from 0.63 to 0.35 and was statistically significant (β = 0.35, *p <* 0.001). Therefore, it was found that wisdom had a partial mediating effect on the relationship between lifestyle and healthy aging, and the significance of the mediating effect was tested by the Sobel test and was statistically significant (Z = 5.11, *p <* 0.001) (Figure 3). 

## 4. Discussion

This study examined the mediating effects of social networks and wisdom on the relationship between lifestyle and healthy aging among community-dwelling older adults with chronic diseases. 

The subjects of this study were elderly people with chronic diseases, and when the general characteristics were examined, the average number of chronic diseases was 2.13, and the chronic diseases were hypertension, hyperlipidemia, arthritis, and diabetes. This is similar to the results that showed that the average number of chronic diseases among the elderly in Korea was 2, and hypertension, hyperlipidemia, arthritis, and diabetes were high [8,35], so the importance of health care for chronic diseases for the elderly is emerging. The Korea Centers for Disease Control and Prevention published a report on the status and issues of chronic diseases in 2021 [36], suggesting that the independence of the elderly decreases and their quality of life decreases when chronic diseases become more severe. Therefore, an Internet site on the prevention and continuous management of chronic diseases by disease is being established and utilized by the public, but due to the lack of computer literacy among the elderly, accessibility and utilization are low, so the support of community experts is needed for more publicity and practical utilization, and it is necessary to activate geriatric health management, including information provision and management plans for chronic diseases of the elderly, centered on the heads of health clinics responsible for primary health care in the community [37]. 

The study’s wisdom was rated at 3.45 out of 5, followed by emotional and interpersonal relationships with 3.74 points, refining and balancing with 3.52, a positive attitude toward life with 3.50 points, and cognitive competence with 3.27. This level indicates the need for efforts to moderately enhance wisdom. Among the sub-areas of wisdom, sympathetic interpersonal relationships demonstrate positive interest and affection for others, as well as cooperative conflict resolution abilities in situations of conflict. This finding mirrors the concept of a “we” relationship-oriented culture in Koreans [21,32], which also garnered the highest score in this study. Refining and balancing involve recognizing and regulating one’s inner emotions, as well as achieving internal integration and balance [21]. A positive life attitude involves living with an understanding of and commitment to a defined mission while also deriving enjoyment from life through the pursuit of goals, visions, and a life philosophy that encompasses the future. Cognitive competence pertains to accurate and profound perceptions, insights, and sound judgments about life [21]. This competency significantly influences life satisfaction and well-being [38,39]. Interestingly, cognitive competence received the lowest score in this study. Cognitive competence, that is, the enhancement of accurate perception and insight into life, necessitates training and educational programs. 

The healthy aging of the subjects was 3.56 out of 5, and the subdomains were physical health, cognitive-mental health, and social support health, of which the cognitive-mental health domain was the lowest, with a score of 3.49. Kim and Seo [4] investigated the healthy aging of older adults with chronic diseases and found a score of 3.41, and Han [40] found a score of 3.37 in a study of older adults living in similar environments and regions, which is similar to this study. This means that the degree of healthy aging of the elderly is evaluated as moderate, so it is necessary to explore strategies to make the elderly spend their old age more healthy. Healthy aging requires the elderly to recognize themselves as constantly changing and adapting, to be active and engaged in daily life, and to continuously evaluate and redefine themselves [3], so healthcare providers should encourage and manage it. In particular, the cognitive and mental health subdomains of healthy aging were somewhat low, so it is necessary to identify psychological health status and make efforts to resolve negative psychological states, such as stress and depression, to improve this area. 

Healthy aging was highly positively correlated with lifestyle habits, social networks, and wisdom of the subjects, and the healthier the lifestyle habits, the higher the degree of social network and wisdom, and the higher the degree of healthy aging. This is supported by the findings that the better the lifestyle of the elderly, the more successful they will be in their later years [10], that the higher the social support and the wider the social network, the better the health preservation and the more successful the later years [13,14], and that the wiser the elderly, the more successful they will be in aging [18,21,22,25]. Healthy lifestyle habits can delay physical aging and play an important role in the healthy living of older adults, including the prevention of geriatric diseases. These habits include a balanced and nutritious diet, rest and sleep, stress management, alcohol consumption, and exercise. Good lifestyle habits are associated with good health, and the cumulative effects of these habits are effective in maintaining good health as we age [10]. It can ensure a healthy old age. Basaraba [41] also stated that people who are healthy and active in lifestyle habits, such as diet, sleep, stress management, drinking habits, and exercise habits, can have a younger biological age than their actual age, which can lead to a healthy old age. In particular, a balanced diet and regular exercise habits help prevent obesity and longevity, and exercise participation leads to expanded opportunities for social interaction and improved quality of life [42], so it is necessary to effectively improve community resources and provide expert management and support for healthy lifestyles. 

The perception of a social network can lead to a sense of security and trust in oneself, leading to a sense of subjective well-being [14]. An elderly person’s social network of relationships is the result of an accumulation over the life course through the establishment of kinship relationships, such as spouse, children, siblings, and non-kinship relationships, such as friends and neighbors. It is not only a means of securing identity through exchanges with others but also has a cumulative multifaceted effect on the daily life of the elderly and positively affects the quality of life in later life [37,43,44], so it is important to secure a social network. To secure social networks, it is necessary to encourage social activities such as occupational and religious activities and lifelong learning activities for the elderly. This helps them to acquire knowledge or information necessary for social adaptation and effective functioning in society. It also helps to reduce feelings of alienation [45] and activate communication and relationship formation with others [46]. Furthermore, the higher the wisdom of the elderly, the more successful they perceived aging, and it was found to be a major factor in successful aging. Counseling and education are needed to increase the wisdom needed to see the lives of the elderly in an integrated manner [25]. 

Next, we found a partial mediating effect of social networks on the relationship between the subjects’ lifestyles and healthy aging. The basics of health care for the elderly are to acquire healthy lifestyle habits in daily life [10]. Efforts should be made to increase exercise habits and stress management habits, which showed low scores among the lifestyle habits of the subjects in this study. Lifestyle habits are a major factor that determines more than 50–60% of an individual’s health status, and it has important relevance in creating a healthy old age by influencing health conditions or diseases [47]. It is also highly related to healthcare utilization and mortality in the elderly [48,49], so lifestyle habits are an issue that should be considered for healthy aging in the elderly. The importance of social networks has emerged in research on the variables that may contribute to healthy aging. Along with lifestyle behaviors, such as good eating habits, maintaining a well-developed social network is critical to the health of older adults [50]. Good habits have been shown to create desirable interpersonal relationships, increase subjective satisfaction, and generate positive emotions [12]. In addition, there was a partial mediating effect of social networks on the relationship between health status and health-related quality of life in the elderly [51], which supported the findings of this study, as social networks can improve quality of life and maintain a healthy old age [51]. In addition, Han [40] found that leisure participation of the elderly was a major influence on healthy aging, but the higher the frequency of leisure participation, the stronger the social networks and the better the quality of life, so it is necessary to improve the social network to spend a healthy old age. 

Older adults become more vulnerable as they age, exposed to deteriorating physical functioning, the stress of the death of a significant other, and changes in social roles and financial challenges, and social support can help them stay positive and improve their ability to cope. Older adults’ social networks consist of activities at home, self-development, and participation in social activities, with family relationships being a key and primary part of their social network. The more active they are at home, the less role loss and decline they experience as they age. Self-development of older adults can help them adapt to new social changes and function effectively in society, providing opportunities to acquire knowledge, information, and skills and enabling them to communicate and relate effectively. In addition, participation in social activities can maintain close relationships with friends and neighbors, delay aging, and redefine the meaning of life [31,52]. Thus, social networks may play a mediating role in the process by which lifestyle habits influence healthy aging. 

Next, wisdom was found to have a partial mediating effect on the relationship between subjects’ lifestyles and healthy aging. In the study of the elderly, wisdom was related to lifestyle behaviors, such as practicing exercise, not drinking alcohol, and eating a regular diet. The wisdom of elderly individuals engaged in leisure activities (such as cultural and arts education and exercise) significantly impacted successful aging. Notably, active investment of substantial time in leisure activities heightened the influence of wisdom, with wisdom fostered through this approach holding a particularly significant role in achieving successful aging [53]. Wisdom plays a crucial role in shaping a meaningful life identity for the elderly as they reflect upon the significance of their past lives [22,54]. The process of realizing life’s meaning or purpose involves aligning wisdom and experience through lifestyle habits, such as engaging in leisure activities, and channeling them into positive strategies through self-assurance and lived experience [53]. The elderly can attain successful aging by practicing regular and appropriate exercise while exercising wisdom [22]. 

Wisdom had a partial mediating effect on the relationship between health promotion behavior and health preservation in daily life [55,56]. These findings underscore the importance, meaning, and purpose of wisdom in the lives of the elderly, significantly impacting the process of positive aging [16,32]. 

In the realm of wisdom, cognitive competence, positive life attitudes, and empathetic interpersonal relationships played pivotal roles in establishing a successful and healthy retirement. As a result, these findings substantiate the study’s conclusions by highlighting the substantial impact of lifestyle habits, including daily physical activities, on successful aging. Moreover, the wisdom demonstrated by the elderly contributes to heightened self-esteem, improved mental health, and enhanced life satisfaction [53]. Additionally, positive life events play a crucial role in the cultivation of wisdom [57]. It can be stated that the events and habits encountered in our daily lives have an impact on wisdom. 

Furthermore, higher levels of wisdom are associated with better health and successful aging, and wisdom has been found to be a major factor in health preservation and successful aging [22,58]. Our results support this finding. The subdomains of wisdom, empathic emotion, self-reflection, and life experience, were all positively correlated with successful aging, suggesting that strategies to increase wisdom are ultimately necessary to achieve successful aging by guiding the maturation and development of older adults through a healthy aging process [16]. In addition, wisdom is an influential factor in the psychological well-being and life satisfaction of older adults, and the higher the level of wisdom, the happier the life [38]. Life satisfaction was associated with better adaptation and psychological well-being [21]. The wisdom of the elderly increases their capacity for self-care and ultimately leads to a successful life with a healthy old age [59]. In addition, a wise person has experience and competence in real-life problems and is rich in social skills and knowledge, as well as transcendent personal qualities [60]. Therefore, wisdom can help older adults realize the meaning and purpose of life, overcome negative changes and physical and psychological losses caused by aging, and make good use of psychological adaptive mechanisms, leading to healthy aging [39]. In particular, wisdom is an important concept for older adults because it is accumulated and increased through various life experiences and can be enhanced in old age [21,38]. 

Wise older adults have been shown to have good lifestyle habits in their daily lives, such as regular eating habits, no smoking and drinking, high sleep satisfaction, and adequate exercise, and wisdom has been reported to influence successful aging [22], so it is likely that healthy lifestyles in older adults are related to wisdom, and that older adults who live wise lives will achieve successful aging through the process of healthy aging. In particular, wisdom is understood as an integration of cognition, emotion, and intuition. It is meaningful when it occurs together in a given context interdependently through empathic emotions, self-reflection, and life-completion experiences [16] in the course of life [19]. Therefore, to increase the wisdom of the elderly, cognitive education and social activities should be promoted to enhance their ability to engage in continuous self-examination and self-awareness, accept life changes with problem-solving and insight, and find meaning in life in harmonious relationships with others [25,54]. 

Therefore, it is necessary to develop interventions to enable the elderly with chronic diseases to have a healthy lifestyle, increase their social network, and have wise life experiences in order to maintain a healthy old age. The results of this study have helped us recognize the significance of wisdom and social networks. Consequently, these findings can serve as fundamental data for guiding future studies that utilize these variables to enhance the lifestyles of the elderly and promote healthy retirement. 

As a limitation of the study, the participants of this study were elderly people in a selected area, so caution should be taken in interpreting the results. The research focused on lifestyle, wisdom, and the underlying social networks are currently inadequate for promoting healthy aging among the elderly. Specifically, there exists a need for a more comprehensive exploration of wisdom and subregion. Research on ways to develop wisdom for the elderly is needed. Furthermore, qualitative research concerning the parameters of this study, namely wisdom and social networks, is also essential. It is advisable to conduct iterative research by expanding both the study’s participant pool and geographical coverage.

## 5. Conclusions

This study was conducted to determine the mediating effects of social networks and wisdom on the relationship between lifestyle and healthy aging in older adults with chronic diseases and to provide a basis for developing intervention programs to improve social networks and wisdom. The results showed that social networks and wisdom had a partial mediating effect. In order to enable the elderly with chronic diseases to lead a healthy lifestyle and live a healthy old age in the face of an ultra-elderly society, it is necessary to develop and apply intervention programs that mediate social networks and wisdom to help them manage their social networks and live a wise life. 

## Figures and Tables

**Table 1:** General characteristics of the participants and the differences in healthy aging according to general characteristics. (*n* = 120).

Variables	Classification	*n*	%	Healthy Aging		t/F	*p*-Value Scheffe’s Test
				Mean	SD		
Age (year)	65–69 ^a^	66	55.0	3.66	0.54	3.76	0.026
	70–79 ^b^	29	24.2	3.57	0.55		a > c
	≥80 ^c^	25	20.8	3.29	0.63		
Gender	Male	38	31.7	3.58	0.59	0.28	0.782
	Female	82	68.3	3.55	0.57		
Spouse	Living together (cohabited)	96	80.0	3.64	0.52	3.11	0.002
	Bereaved, divorced, separated, unmarried	24	20.0	3.24	0.68		
Education	Graduated from elementary school or lower ^a^	25	20.8	3.27	0.68	9.08	<0.001
	Graduated from middle school or high school ^b^	62	51.7	3.51	0.51		a, b < c
	Graduated from university or higher ^c^	33	27.5	3.87	0.47		
Religion	No	60	50.0	3.59	0.58	0.50	0.619
	Yes	60	50.0	3.53	0.58		
Job	No	69	57.5	3.52	0.58	−0.80	0.424
	Yes	51	425	3.61	0.56		
Kinds of jobs	Professional or educational occupation	14	23.7	3.83	0.55	2.10	0.111
	A service profession	14	23.7	3.51	0.33		
	Self-employment	11	18.6	3.32	0.69		
	Others	20	34.0	3.66	0.54		
The number of diseases	1 ^a^	60	50.0	3.73	0.46	8.65	<0.001
(number)	2–3 ^b^	40	33.3	3.50	0.47		a > c
	≥4 ^c^	20	16.7	3.17	0.84		
Economic level	Low or medium	96	80.0	3.50	0.53	−2.45	0.016
	High	24	20.0	3.81	0.70		

SD = standard deviation.

**Table 2:** Degree of lifestyle habits, social networks, wisdom and healthy aging of participants. (*n* = 120).

Variables	Mean	SD	Actual Range
Lifestyle habits	2.84	0.32	1.95–3.67
Dietary habits	2.92	0.40	2.08–4
Resting and sleeping habits	2.83	0.62	1.33–4
Stress management habits	2.71	0.44	1.30–3.80
Drinking habits	3.29	0.45	1.20–4
An exercise habit	2.60	0.67	1–4
Social networks	2.95	0.69	1.33–4.80
Social participation activities	2.75	0.85	1–5
Self-development activities	3.02	1.02	1–5
Domestic activities	3.09	0.71	1–4.8
Wisdom	3.45	0.60	1.09–4.81
Cognitive competence	3.27	0.71	1.19–4.94
Refining and balancing	3.52	0.63	1–5
A positive attitude toward life	3.50	0.66	1.1–5
Empathetic interpersonal relationships	3.74	0.72	1–5
Healthy aging	3.56	0.58	1.70–4.90
Physical health	3.54	0.65	1.83–5
Cognitive and mental health	3.49	0.77	1–5
Social support health	3.62	0.66	1–5

SD = standard deviation.

**Table 3:** Relationships between lifestyle habits, social networks, wisdom, and healthy aging of participants.

Variables	Lifestyle Habits R (*p*)	Social Networks R (*p*)	Wisdom R (*p*)	Healthy Aging R (*p*)
Lifestyle habits	1			
Social networks	0.43 (<0.001)	1		
Wisdom	0.51 (<0.001)	0.62 (<0.001)	1	
Healthy aging	0.63 (<0.001)	0.65 (<0.001)	0.73 (<0.001)	1

**Table 4:** The mediating effect of social networks in the relation between lifestyle habits and healthy aging in participants.

Variables	B	SE	β	t (*p*)	R^2^	Adj. R^2^	F (*p*)
Step 1: Lifestyle habits → Social networks	0.92	0.18	0.43	5.15 (<0.001)	0.184	0.177	26.54 (<0.001)
Step 2: Lifestyle habits → Healthy aging	1.12	0.13	0.63	8.83 (<0.001)	0.398	0.393	77.92 (<0.001)
Step 3: Lifestyle habits, social networks → Healthy aging	0.77 0.39	0.12 0.06	0.43 0.46	6.46 (<0.001) 0.95 (<0.001)	0.574	0.566	78.75 (<0.001)

B = non-standardized beta, SE = standard error, β = standardized beta.

**Table 5:** The mediating effect of wisdom in the relation between lifestyle habits and healthy aging in participants.

Variables	B	SE	β	t (*p*)	R^2^	Adj. R^2^	F (*p*)
Step 1: Lifestyle habits → Wisdom	0.94	0.15	0.51	6.39 (<0.001)	0.257	0.251	40.89 (<0.001)
Step 2: Lifestyle habits → Healthy aging	1.12	0.13	0.63	8.83 (<0.001)	0.398	0.393	77.92 (<0.001)
Step 3: Lifestyle habits, Wisdom → Healthy aging	0.62 0.53	0.12 0.06	0.35 0.55	5.33 (<0.001) 8.45 (<0.001)	0.626	0.620	97.88 (<0.001)

B = non-standardized beta, SE = standard error, β = standardized beta.

## Data Availability

The data underlying this article will be shared upon reasonable request from the corresponding author.

## Appendix A

The following is a question about wisdom. Please mark V where it matches your opinion.

**Table A1:** Wisdom Instrument.

Items	Not True at All	Mostly Untrue	Somewhat True	Generally True	Absolutely True
I am able to make accurate and swift judgments.					
My problem-solving skills are excellent.					
I am skilled at persuading others.					
I possess keen insights and a strong grasp of the nature of things.					
I can apply knowledge flexibly in my daily life.					
I have the ability to express my thoughts effectively.					
I generate unique ideas that are beyond the reach of others.					
I possess foresight and an ability to anticipate future trends.					
I am adept at examining situations from various perspectives.					
I possess the capability to comprehend the bigger picture.					
I adapt flexibly to different situations.					
I possess the ability to provide constructive criticism.					
My keen sense of observation allows me to perceive details others might miss.					
I am skilled at effectively using rewards and punishments to manage people.					
I possess a deep understanding of the underlying logic of the world.					
I have a strong intuition for understanding people.					
Maintaining composure in any circumstance is a strength of mine.					
I am capable of stepping back from problems to gain perspective.					
Self-control is one of my strengths.					
I maintain a clear distinction between the public and private aspects of my life.					
I am not biased in any aspect of my life and excel at balancing.					
I maintain my composure.					
I avoid repeating mistakes.					
I pursue significant goals rather than fixating on minor interests.					
I treat people without prejudice or stereotypes.					
I avoid excessive greed.					
I carefully consider my actions before taking them.					
I have clear goals and visions for the future.					
I contemplate the values and goals of life seriously.					
I am always striving for self-improvement and personal growth.					
I derive enjoyment from my job.					
I have developed my own life philosophy.					
I am familiar with and actively practice the missions assigned to me.					
I find pleasure in my personal life.					
I impart lessons and insights to others.					
I frequently engage in self-reflection.					
I understand the finite nature of life.					
I possess the ability to view situations from another person’s perspective.					
I comprehend and empathize with the emotions of others.					
I demonstrate respect for others.					
I avoid imposing my argument unilaterally.					
I readily admit my faults and mistakes.					
I possess an open mind that embraces the shortcomings of others.					
